# Combination of Lenvatinib and Pembrolizumab Is an Effective Treatment Option for Anaplastic and Poorly Differentiated Thyroid Carcinoma

**DOI:** 10.1089/thy.2020.0322

**Published:** 2021-07-08

**Authors:** Christine Dierks, Jochen Seufert, Konrad Aumann, Juri Ruf, Claudius Klein, Selina Kiefer, Michael Rassner, Melanie Boerries, Andreas Zielke, Paul la Rosee, Philipp Tobias Meyer, Matthias Kroiss, Christian Weißenberger, Tilmann Schumacher, Patrick Metzger, Harald Weiss, Constantin Smaxwil, Katharina Laubner, Justus Duyster, Nikolas von Bubnoff, Cornelius Miething, Oliver Thomusch

**Affiliations:** ^1^ Department of Hematology and Oncology, KIM IV, Faculty of Medicine, University Halle-Wittenberg, Halle, Germany.; ^2^ Department of Hematology and Oncology, University of Freiburg, Freiburg, Germany.; ^3^ Division of Endocrinology and Diabetology, Department of Medicine II, University of Freiburg, Freiburg, Germany.; ^4^ Institute of Pathology, University of Freiburg, Germany.; ^5^ Department of Nuclear Medicine, University of Freiburg, Germany.; ^6^ German Cancer Research Center (DKFZ), Heidelberg, Germany.; ^7^ Comprehensive Cancer Center Freiburg (CCCF), University Medical Center, University of Freiburg, Freiburg, Germany.; ^8^ Institute of Medical Bioinformatics and Systems Medicine and Institute of Molecular Medicine and Cell Research; Faculty of Medicine, University of Freiburg, Freiburg, Germany.; ^9^ Outcomes Research Unit, Department of Endocrine Surgery, Endocrine Center Stuttgart, Diakonie Klinikum Stuttgart, Stuttgart, Germany.; ^10^ Klinikum Villingen-Schwenningen, Hämatologie/Onkologie, Villingen-Schwenningen, Germany.; ^11^ German Cancer Consortium (DKTK), Partner Site Freiburg, Freiburg, Germany.; ^12^ Division of Endocrinology/Diabetology, Department of Internal Medicine, University Hospital Würzburg, Würzburg, Germany.; ^13^ Comprehensive Cancer Center Mainfranken, University of Würzburg, Würzburg, Germany.; ^14^ Zentrum für Strahlentherapie, Freiburg, Germany.; ^15^ Praxis für Nuklearmedizin, Freiburg, Germany.; ^16^ Faculty of Biology, University of Freiburg, Freiburg, Germany.; ^17^ Eisai GmbH, Frankfurt/Main, Germany.; ^18^ Department of Hematology/Oncology, University of Luebeck, Luebeck, Germany.; ^19^ Department of General and Visceral Surgery, University Hospital Freiburg, Freiburg, Germany.

**Keywords:** anaplastic thyroid cancer, ATC, lenvatinib, PDTC, pembrolizumab, poorly differentiated thyroid cancer

## Abstract

***Background:*** Anaplastic thyroid carcinoma (ATC) and metastatic poorly differentiated thyroid carcinomas (PDTCs) are rare aggressive malignancies with poor overall survival (OS) despite extensive multimodal therapy. These tumors are highly proliferative, with frequently increased tumor mutational burden (TMB) compared with differentiated thyroid carcinomas, and elevated programmed death ligand 1 (PD-L1) levels. These tumor properties implicate responsiveness to antiangiogenic and antiproliferative multikinase inhibitors such as lenvatinib, and immune checkpoint inhibitors such as pembrolizumab.

***Patients and Methods:*** In a retrospective study, we analyzed six patients with metastatic ATC and two patients with PDTC, who received a combination therapy of lenvatinib and pembrolizumab. Lenvatinib was started at 14–24 mg daily and combined with pembrolizumab at a fixed dose of 200 mg every three weeks. Maximum treatment duration with this combination was 40 months, and 3 of 6 ATC patients are still on therapy. Patient tumors were characterized by whole-exome sequencing and PD-L1 expression levels (tumor proportion score [TPS] 1–90%).

***Results:*** Best overall response (BOR) within ATCs was 66% complete remissions (4/6 CR), 16% stable disease (1/6 SD), and 16% progressive disease (1/6 PD). BOR within PDTCs was partial remission (PR 2/2). The median progression-free survival was 17.75 months for all patients, and 16.5 months for ATCs, with treatment durations ranging from 1 to 40 months (1, 4, 11, 15, 19, 25, 27, and 40 months). Grade III/IV toxicities developed in 4 of 8 patients, requiring dose reduction/discontinuation of lenvatinib. The median OS was 18.5 months, with three ATC patients being still alive without relapse (40, 27, and 19 months) despite metastatic disease at the time of treatment initiation (UICC and stage IVC). All patients with long-term (>2 years) or complete responses (CRs) had either increased TMB or a PD-L1 TPS >50%.

***Conclusions:*** Our results implicate that the combination of lenvatinib and pembrolizumab might be safe and effective in patients with ATC/PDTC and can result in complete and long-term remissions. The combination treatment is now being systematically examined in a phase II clinical trial (Anaplastic Thyroid Carcinoma Lenvatinib Pembrolizumab [ATLEP]) in ATC/PDTC patients.

## Introduction

Anaplastic thyroid carcinoma (ATC) is a rare disease with an extremely high mortality rate and a 10-year survival below 5% (1). ATCs grow very fast, infiltrate into cervical structures, such as the esophagus, trachea, or blood vessels, and metastasize to the lung, brain, and bone. Even with extensive multimodal therapy (surgery, external beam radiotherapy, and chemotherapy), the median overall survival (OS) is only 3–5 months (1–3). ATC frequently arises from differentiated thyroid carcinoma (DTC) but can also emerge *de novo*. Typical molecular features of ATC are mutations in *TP53* (54–64%), *TERT* (61%), *KRAS/NRAS* (28–43%), *BRAF* (18–28%), *NF1*, *NF2*, *PI3K*, *PTEN*, and genes in the *WNT* signaling pathway (4–9). BRAF/MEK inhibitor combinations (dabrafenib/trametinib) are approved for *BRAF^V600E^*-mutated ATCs and have significantly enhanced survival and has high treatment response (overall response rate [ORR] 69%) (10,11). All other locally advanced or metastatic ATCs (about 75%) are individually treated with carboplatin, paclitaxel, or doxorubicin containing chemotherapeutic regimens with very modest ORR (10–20%) lasting only for a few months (progression-free survival [PFS] 3–4 months) (12–15). Poorly differentiated thyroid carcinoma (PDTC) has a more favorable prognosis, with some patients being partially responsive to radioiodine therapy (RIT), but 10-year survival is also below 10%.

Compared with DTC, ATC/PDTC are characterized by an elevated tumor mutation burden, higher programmed death ligand 1 (PD-L1) levels, and increased neoangiogenesis (VEGFR/FGFR signaling) (7,16–22), suggesting that they may be sensitivity to immune checkpoint and neoangiogenesis inhibitors.

Lenvatinib is an antiangiogenic (VEGFR 1–3/FGFR 1–4) and antiproliferative (RET/PDGFR) tyrosine kinase inhibitor (TKI), which is approved for DTC refractory to radioiodine treatment (23). PFS in DTC is 19.4 months (23–26), but it is reduced to 14.8 months in PDTC, and all patients ultimately develop treatment resistance or treatment intolerance (26). In ATC, the PFS is even shorter (5.6–7.4 months) with variable ORR between 17.4% and 43%, depending on the initial tumor stage (27–29).

Pembrolizumab is an immune checkpoint inhibitor targeting PD-1 on immune cells. Response to pembrolizumab treatment is associated with elevated expression of PD-L1 or high tumor mutational burden (TMB) (30–34). DTC have low TMB and low PD-L1 expression (CPS/TPS) and hence have low response rates to immune checkpoint inhibitor treatments (ORR 9%) (35). In ATC with higher PD-L1/TMB, the effect of immune checkpoint inhibitors is still low (ORR <10%) (36,37), as the slow response to treatment (>8 weeks) cannot keep pace with the aggressive tumor growth.

*In vitro* studies and mouse experiments indicate synergism between lenvatinib and pembrolizumab (38). Lenvatinib not only blocks tumor neoangiogenesis and proliferation but also enhances immune cell invasion into the tumor and therefore fosters immune responses induced by immune checkpoint inhibitors (38). Furthermore, phase II trials with the lenvatinib/pembrolizumab combination in patients with extensively pretreated metastatic solid tumors (renal, ovarian, and endometrial carcinoma) not only showed acceptable toxicity but also very promising response rates (84% partial response [PR], 16% stable disease [SD]). Phase II/III trials for endometrial, head and neck, and hepatocellular carcinoma validated the high efficacy and safety for this combination (39–42). In ATC, pembrolizumab treatment was used as a salvage strategy after patients had failed lenvatinib or dabrafenib monotherapy or a dabrafenib/trametinib combination therapy. Pembrolizumab was added to the respective TKI and induced PR rates of 43%, but with a very short PFS of only 2.96 months (43).

In contrast to this study, we directly combined lenvatinib and pembrolizumab as front-line therapy after chemotherapy failure in eight patients with metastatic ATC (*n* = 6) or PDTC (*n* = 2) and examined treatment outcome and biomarkers for this approach.

## Materials and Methods

### Study population and patient characteristics

This is a retrospective, single-center (University Medical Center Freiburg) cohort study of eight patients with metastatic ATC (*n* = 6) or PDTC (*n* = 2) treated with a combination of the multikinase inhibitor lenvatinib and the immune checkpoint inhibitor pembrolizumab (L/P). Patient data were collected from March 2016 to December 2019 (Table 1). ATC/PDTC diagnosis was histopathologically confirmed by the Institute for Pathology, Freiburg, in all patients. Patients had no *BRAF^V600E^* mutation, but numerous other mutations were present (Supplementary Table S1). All patients were pretreated with irradiation, chemotherapy, or RIT (Table 1 and Supplementary Table S2). Adverse events (AEs) were reported according to the National Cancer Institute Common Terminology Criteria for Adverse Events (CTCAE version 5)–GBG (Table 2). The retrospective study was approved by the Institutional Review Board (IRB) of the University Freiburg.

**Table 1.** Baseline Characteristics

Median age at treatment start (range), years	63.5 (49–88)
Sex, *n* (%)	
Men	4 (50)
Women	4 (50)
Performance status, *n* (%)	
ECOG 0	3 (37.5)
ECOG 1	3 (37.5)
ECOG 2	2 (25)
Pathological diagnosis, *n* (%)	
ATC	6 (75)
PDTC	2 (25)
Location of metastases, *n* (%)	
Lung	8 (100)
Bone	2 (25)
Kidney	1 (12.5)
Brain	1 (12.5)
Liver	1 (12.5)
Skin	1 (12.5)
Cervical relapse, *n* (%)	6 (75)
Previous therapy, *n* (%)	
Surgery	8 (100)
Radiation ± chemosensitizing	7 (87.5)
Chemotherapy	6 (75)
RIT	2 (25)

ATC, anaplastic thyroid carcinoma; ECOG, Eastern Cooperative Oncology Group; PDTC, poorly differentiated thyroid carcinoma; RIT, radioiodine therapy.

**Table 2.** Adverse Events According to the Common Terminology Criteria for Adverse Events Version 5.0

Event	Grade I/II (%)	Grade III/IV (%)
Total	8/8 (100)	3/8 (36)
Hypertension	5/8 (63)	
Fatigue	2/8 (25)	1/8 (13)
Anorexia	2/8 (25)	2/8 (25)
Oral mucositis	2/8 (25)	
Joint/muscle pain	1/8 (13)	
Hand–foot syndrome	1/8 (13)	
Diarrhea	1/8 (13)	
Proteinuria	1/8 (13)	
Abdominal pain		1/8 (13)
Cervical bleeding		1/8 (13)

Data are given as totals and %. Events reported are listed in descending frequency of columns for grade I/II and grade III/IV. A patients with several multiple occurrences of an adverse event is counted only once with the highest grade. A patient with multiple adverse events is counted only once in the total row.

### Treatment strategy

Patients started with lenvatinib 24 mg oral daily if the body weight (BW) was more than 80 kg, or 20 mg for BW less than 80 kg. Pembrolizumab infusions were started in between 1 and 4 weeks after initiation of lenvatinib at a fixed dose of 200 mg total every 3 weeks. Dose for lenvatinib was stepwise reduced upon occurrence of side effects according to clinical judgment. Lenvatinib was given at least for 1 year and was then stopped in patients with confirmed complete response (CR). Pembrolizumab was given for up to 40 months. It will be continued in all patients reaching a CR for two more years.

### Response assessment

**FIG. 1.**
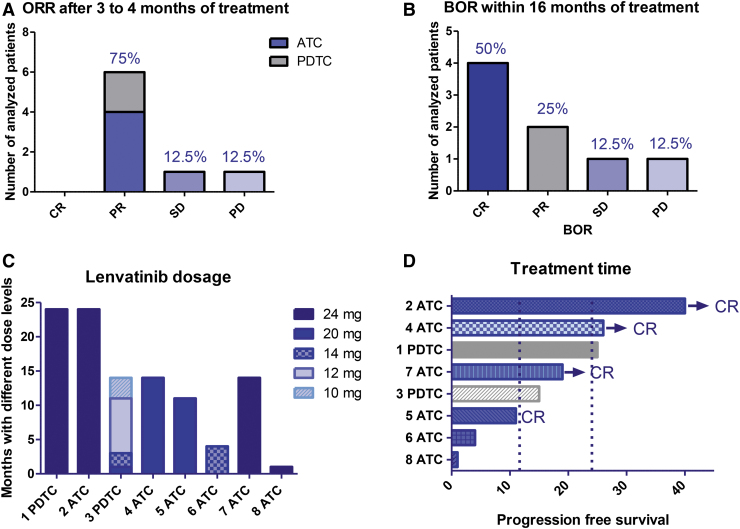
(**A**) ORR after 3–4 months of treatment, 6/8 PR, 1/8 SD, 1/8 PD. (**B**) BOR within 16 months of treatment. (**C**) Lenvatinib dosage and changes over time. (**D**) Treatment duration and ongoing treatment. Patients in CR. BOR, best overall response; CR, complete response; ORR, overall response rate; PD, progressive disease; PR, partial response; SD, stable disease.

Radiology assessment including cervical, chest, and abdominal computed tomography (CT) scans was performed before treatment and then every 3–4 months. Response to therapy was determined centrally using the RECIST 1.1 criteria (Fig. 1A and Supplementary Table S3). Positron emission tomography (PET)–CT using [^18^F] fluorodeoxyglucose (FDG) (PET/CT) was performed before treatment, and after 12–16 months of treatment to confirm CRs (the European Organisation for Research and Treatment of Cancer [EORTC] response criteria for PET). In case of persistent lesions without tracer uptake on PET/CT (PR according to the RECIST 1.1 [CT scan], but CR according to the EORTC criteria for PET), lesions were surgically removed (1/8) to confirm the absence of viable tumor tissue (pathological CR criteria, Supplementary Table S4).

The efficacy of the combination treatment was assessed by ORR 3–4 months after starting treatment, best overall response (BOR) (Fig. 1B), PFS, and OS. PFS was defined as the time elapsed between starting treatment and progression or death, whichever occurred first. OS was defined as the time between starting treatment and death.

### Molecular testing and immunohistochemistry

Molecular testing by whole-exome sequencing (WES) was performed from formalin-fixed and paraffin-embedded tissue specimens at the Deutsches Krebsforschungszentrum Heidelberg (DKFZ) as described previously (44,45). PD-L1 status was determined by immunohistochemistry (antibody clone SP263; Ventana) in tumor tissue specimens obtained at initial diagnosis before treatment. The tumor proportion score (TPS) was determined as proportion of PD-L1-positive tumor cells of 100 tumor cells. The combined proportion score (CPS), as amount of PD-L1-positive tumor and immune cells within 100 tumor cells, was also determined centrally at the Department of Pathology of the University Medical Center Freiburg.

### Statistical analysis

Kaplan–Meier curves were used for OS and PFS. Descriptive statistics were used to summarize patients' characteristics and AEs. Statistical analysis was performed using IBM SPSS Statistics version 25.

## Results

Eight patients with metastatic ATC (*n* = 6) or PDTC (*n* = 2) were treated with a combination of lenvatinib and pembrolizumab after failing chemotherapy, irradiation, or RIT (Table 1 and Supplementary Table S2). All the 8 patients had been extensively pretreated with surgery (8/8), local cervical external beam radiation therapy (6/8), RIT (2/2), local cerebral irradiation therapy (1/8), or systemic chemotherapy alone (6/8) (carboplatin/paclitaxel [4/8], cisplatin/paclitaxel [1/8], cisplatin/doxorubicin [1/8], and paclitaxel only [1/8]) (Table 1 and Supplementary Table S2). The median patient age was 66.4 years. At the beginning of treatment with lenvatinib/pembrolizumab, all patients had stage IVC. All patients had lung metastasis (8/8), and 2 of 8 skeletal metastasis, 1 of 8 liver metastasis, 1 of 8 kidney metastasis, 1 of 8 skin metastasis, and 1 of 8 brain metastasis. Six of eight patients also had progression of their local tumor. Eastern Cooperative Oncology Group (ECOG) performance status ranged from 0 to 2, with 3 ECOG 0 (38%), 3 ECOG 1 (38%), and 2 ECOG 2 (25%). Molecular diagnostics showed that none of the patients had a *BRAF^V600E^* mutation, but numerous other mutations typical for ATC/PDTC were detected and are listed in Supplementary Table S1.

### Treatment regimen and AEs

Lenvatinib was started at a daily dose of 24 mg in 5 of 8 patients (BW more than 80 kg) and 20 mg in 2 patients with bodyweight less than 80 kg. An 88-year-old woman started on 14 mg/day (Fig. 1C and Table 3). Pembrolizumab treatment was initiated after a median of 2.7 weeks (ranging from 1 to 4 weeks after starting lenvatinib) and was given i.v. at a fixed dose of 200 mg every 3 weeks. In general, the therapy was well tolerated with the predominant grade II to IV AEs (AEs according to the CTCAE) being hypertension (5/8), fatigue (4/8), weight loss/anorexia (3/8), oral mucositis (2/8), diarrhea (2/8), joint/muscle pain (1/8), and hand–foot syndrome (1/8) (Table 2). The lenvatinib dose was reduced stepwise upon occurrence of intolerable side effects from 24 to 20, 14, 12, and 10 mg/day (Fig. 1C). Most side effects resolved after reducing the dose of lenvatinib, but in two patients, lenvatinib-induced AEs required treatment discontinuation (patients 3 and 6, Table 3). One grade IV serious adverse event (SAE) with a lethal cervical bleeding occurred after removal of the tracheostomy in patient 5 despite being in complete remission (Tables 2 and 3).

**Table 3.** Patient History

Patient	Entity	Age, years	Prior treatment	ORR in 3/4 months	BOR CT+PET	PFS, months	OS, months	Adverse events (CTCAE in grade °)	Median dose lenvatinib, mg/day	Current therapy/ outcome
1	PDTC	63	S Rx C/T	PR	PR	25	27	Hypertension °II Fatigue °II Anorexia °I Oral mucositis °I	24	PD/death after stopping lenvatinib due to a knee surgery
2	ATC	76	S Rx C/T	PR	CR	40	40	Hypertension °II Anorexia °II	24	Alive, CR after 12 months lenvatinib/pembrolizumab, now without treatment
3	PDTC	49	S RIT C/T	PR	PR	15	23	Anorexia °III Abdominal pain °III	14	PD/death, lenvatinib on/off due to weight loss/abdominal pain
4	ATC	68	S Rx C/T	PR	CR	26	26	Anorexia °I Loss of appetite °II Proteinuria °I Hypertension °II	20	Alive, CR after 10 months of treatment, now pembrolizumab mono
5	ATC	63	S Rx C/T	PR	CR	11	11	Diarrhea °II Hypertension °II Cervical bleeding °IV	20	CR after 7 months of treatment. Death due to cervical bleeding
6	ATC	88	S Rx C/T	SD	SD	4	7	Diarrhea °II Anorexia °III Proteinuria °II Fatigue °III Oral mucositis °II	14	PD/death after stopping medication due to side effects
7	ATC	64	S RIT Rx	PR	CR	19	19	Hypertension °II Fatigue °II Joint pain °II Hand–foot syndrome °I	24	Alive, CR, lenvatinib/pembrolizumab for 12 months, now pembrolizumab mono
8	ATC	60	S R C/T	PD	PD	1	1	Hypertension °II	24	PD/death due to cervical tumor progression

Patient characteristics including diagnosis, previous therapy (S, RIT, Rx, C/T), patient age, PFS, ORR, BOR, OS, response after 3 months, maximum response, main side effects, median dose lenvatinib in mg/day, dose pembrolizumab, current treatment/outcome. CTCAE grades in roman numbers. Responses were assessed via the RECIST v1.1 radiology assessment and FDG uptake according to the EORTC criteria for PET.

BOR, best overall response; C/T, chemotherapy; CT, computed tomography; CR, complete response; CTCAE, Common Terminology Criteria for Adverse Events; FDG, [^18^F] fluorodeoxyglucose; ORR, overall response rate; OS, overall survival; PD, progressive disease; PET, positron emission tomography; PFS, progression free survival; PR, partial response; Rx, radiation therapy; S, surgery; SD, stable disease.

After four months of treatment, lenvatinib/pembrolizumab was discontinued due to severe weight loss/anorexia (grade III) in the 88-year-old patient (patient 6), and she died due to disease progression three months later. One patient was intolerant to lenvatinib treatment due to grade III abdominal pain and weight loss/anorexia (patient 3). Lenvatinib was reduced from 24 to 20 to 14 and 12 mg/day in a stepwise manner, and after 14 months, lenvatinib/pembrolizumab treatment was discontinued. Two patients received the full dose of lenvatinib 24 mg for 24 months (Fig. 1C). Pembrolizumab was given for up to 40 months and will be continued in all patients reaching a CR for two more years (Fig. 1D).

### Treatment efficacy

Treatment response was first assessed 3–4 months after lenvatinib/pembrolizumab treatment. Six of eight patients had a PR according to the RECIST v1.1 criteria (Fig. 1A). ORR for ATCs was 66% (4/6 PR). The 88-year-old patient (ATC) at 14 mg lenvatinib had SD (patient 6). One ATC patient (patient 8) did not respond to lenvatinib/pembrolizumab treatment combination and died within the first month of treatment due to cervical tumor progression (1/8 progressive disease) (Fig. 1A and Supplementary Data).

BOR changed in four ATC patients from PR to CR within 16 months of treatment (total CR rate 50%, CR rate for ATC 66%) (Fig. 1B). Individual patient history is summarized in the Supplementary Data. ATC patient 2 had a confirmed CR for all target- and nontarget lesions 16 months after lenvatinib/pembrolizumab treatment, and stopped taking lenvatinib after 2 years (24 mg daily for 24 months), but continued pembrolizumab for 16 more months (40 months total). He stopped treatment for 6 months and is still in CR. ATC patient 4 with lung and brain metastases had a PR 12 months after starting treatment, but all lesions in the lung were without FDG uptake (CR according to the EORTC criteria for PET). All lung lesions were surgically removed and showed a pathological CR (no viable tumor cells). Therefore, the patient was judged as having a CR and has discontinued lenvatinib after 1 year of treatment, and continues with pembrolizumab only (26 months total). Patient 7 with a massive neck tumor and lung metastasis had a PET/CT confirmed CR 12 months after starting treatment. He stopped lenvatinib after 15 months and now continues with pembrolizumab only (19 months total). ATC patient 5 had confirmed CR 10 months after treatment start, but unfortunately died due to bleeding complications after removal of the tracheostomy tube (Table 3 and Supplementary Table S2).

**FIG. 2.**
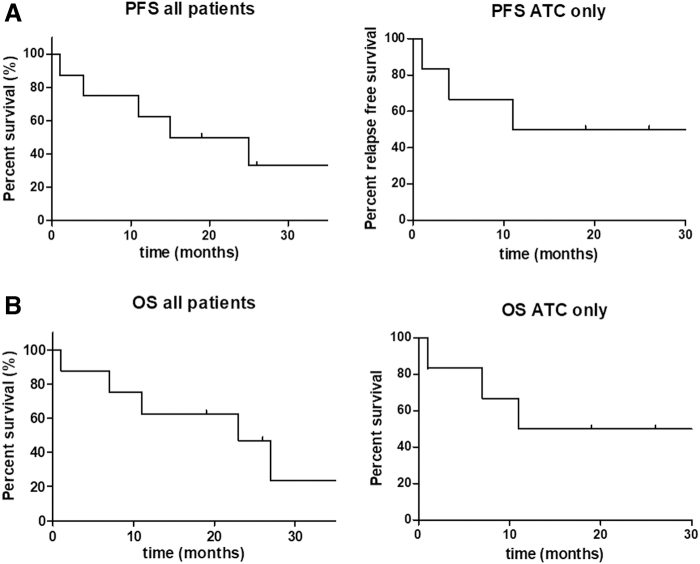
Kaplan–Meier curves for ATCs only and total patients. (**A**) PFS in all patients and ATC only. (**B**) OS in all patients and ATC only. ATC, anaplastic thyroid carcinoma; OS, overall survival; PFS, progression-free survival.

Treatment durations ranged from 1 to 40 months (ATC: 40, 26, 19, 18, 4, and 1 month; PDTC: 25 and 15 months, respectively), with three patients being treated for more than 2 years (Fig. 1D). At the time of data cutoff, 3 of 8 patients (patients 2, 4, and 7, all ATCs) were still alive and on therapy (40, 26, and 19 months). We stopped the treatment of patient 2 after 40 months of treatment, and he is now regularly monitored by CT scans every 3 months. The other patients died due to disease progression (2/6 ATC, 2/2 PDTC) or hemorrhage (grade IV SAE, patient 5, Table 3). ATC patient 8 was resistant to the treatment. ATC patients 1 and 6 died shortly after discontinuation of the lenvatinib treatment caused by lenvatinib intolerance in patient 6 (grade III anorexia), and because lenvatinib had to be discontinued due to an infectious complication after a knee surgery in patient 1 (Table 3). The median PFS for the total cohort was 17.6 months, and the median OS was 19 months (Fig. 2). Data analysis for ATC only showed a median PFS of 16.8 months and a median OS of 17.3 months (Fig. 2).

### Biomarkers

To assess potential predictors of treatment response, we investigated the PD-L1 expression of the tumor cells and macrophages/immune cells. Furthermore, WES was performed in 7 of 8 patients, and sequences were compared to germ line sequences to assess somatic mutations and the TMB. In 1 patient (1/8), targeted sequencing for 11 frequently mutated genes (Supplementary Table S1) was performed due to low material. All tumors were positive for PD-L1 expression with a TPS ranging from 1% to 90%, and 5 of 8 patients with TPS >50% (Table 4). The CPS ranged from 5 to 100 (Table 4). Interestingly, the ATC patient with the lowest TPS (1%) and CPS 5 did not respond to the treatment (patient 8). In contrast, the patients with responses lasting more than two years and those achieving a CR all had PD-L1 TPS >50% (5/8), a CPS >75 (3/8), or a high mutation frequency above 5 mutations per megabase (3/8). The patient (patient 2) with the highest number of mutations (>1400 somatic mutations) has the longest CR duration (30 months).

**Table 4.** Biomarker Analysis

Patient	ORR after 3/4 months (RECIST 1.1)	BOR (CT/MRI/PET-CT)	Response according to PET-CT	TPS, %	CPS	Somatic mutations	TMB (mutations/Mb)
1	PR	PR	PR	50	40	106	13.79
2	PR	CR	CR	60	75	1447	81.87
3	PR	PR	PR	10	10	79	4.08
4	PR	CR	CR	90	100	19	3
5	PR	CR	n.a.	80	100	29	3.3
6	SD	SD	n.a.	60	65	24	3.58
7	PR	CR	CR	5	7	138	5.59
8	PD	PD	n.a.	1	5	n.a.	n.a.

CPS, combined proportion score; MRI, magnet resonance tomography; n.a., not applicable; TMB, tumor mutation burden; TPS, tumor proportion score.

## Discussion

Current treatment options for ATC are limited, and response rates are low and short-lived, with marginal to absent CR (13,14). Only the small proportion of *BRAF^V600E^*-mutated ATC (about 20%) can be effectively treated with a BRAF/MEK inhibitor combination of dabrafenib and trametinib (10,11), but for the other 70–80% of ATCs, no treatment options are approved after chemotherapy failure in most countries.

ATC and PDTC are characterized by a very high proliferation rate and tumor invasiveness, driven by concurrent mutations in several pro-proliferative pathways (RAS, WNT, loss of TP53), and strongly activated VEGF/FGF signaling. PD-L1 and TMB are upregulated, but the response to single immune checkpoint inhibition often comes too late and is overrun by the aggressiveness of the disease (36).

While many kinase inhibitors (sorafenib/pazopanib) failed to show treatment efficacy in ATC (30–32), lenvatinib trials demonstrated some promising effects, with 17.4–43% PRs and up to 50% SD (27–29). CRs were absent and PFS lasted only between 77 days and 7.4 months (27–29). Besides its fast, but short-lived antiproliferative effects, lenvatinib was shown to improve immune responses to immune checkpoint inhibitors in mice. Therefore, by combining both agents, we aimed to use lenvatinib as a fast and effective bridging treatment and immuno-sensitizer for the immune checkpoint inhibitor pembrolizumab in ATC/PDTC patients.

In contrast to single-agent therapy, the combination of lenvatinib and pembrolizumab was highly effective in our treatment cohort. Eight ATC/PDTC patients who had previously been treated with several lines of therapy including chemotherapy, chemoirradiation, and even single immune checkpoint inhibition (1/8) received a combination of lenvatinib and pembrolizumab for a maximum of 40 months. The combination treatment was well tolerated and could be sustained over one year in half of the patients, and even over two years in 37% (3/8) of the patients. Half of the ATC patients (3/6) were still on therapy at data cutoff with no sustained grade III/IV toxicities despite having initially metastatic disease; three patients had confirmed CR by PET/CT and/or histopathologic examination of former metastatic sites. Seventy-five percent of all patients and 66% of the ATC patients reached a PR/CR within 16 months of treatment. The remission rates observed with lenvatinib/pembrolizumab combination in ATC/PDTC are similar to previously published data for other heavily pretreated solid tumors, such as patients with head and neck tumors, endometrial, renal cell, or hepatocellular carcinoma (39–42). As CRs and long-lasting remissions were rarely observed in ATC patients treated with lenvatinib only (27–29), the addition of pembrolizumab is most likely inducing this effect.

In patients who are already resistant to TKI treatment, the addition of pembrolizumab is only partially functional with a PFS of only 2.96 months (43), which indicates that the up-front combination of lenvatinib and pembrolizumab might be much more effective than using these drugs sequentially.

TMB and PD-L1 expression levels (TPS/CPS) are independent biomarkers for response to immune checkpoint inhibitors in solid tumors (30,46,47). In our analysis, patients reaching a CR or those who had long-term remission over two years all had a TPS above 50%, a CPS higher than 75, and/or a TMB >5/MB, indicating that those may be biomarkers for response to lenvatinib/pembrolizumab treatment in ATC/PDTC.

In general, the combination of lenvatinib and pembrolizumab was well tolerated even in the elderly patients with a higher ECOG performance status. Results are encouraging with an ORR of 75%, including a CR rate of 50% (66% in ATCs) and responses over 2 years. Therefore, the treatment results and biomarkers will be further evaluated in a phase II clinical trial with lenvatinib and pembrolizumab in ATC/PDTC patients (ATLEP trial, Anaplastic Thyroid Carcinoma Lenvatinib Pembrolizumab, EudraCT No. 2017-004570-34).

## Supplementary Material

Supplemental data

Supplemental data

Supplemental data

Supplemental data

Supplemental data
