# Supplementary material for: Combination of Lenvatinib and Pembrolizumab Is an Effective Treatment Option for Anaplastic and Poorly Differentiated Thyroid Carcinoma
Source: Thyroid. 2021 Jul 8;31(7):1076–85. doi: 10.1089/thy.2020.0322 (PMC8290324; doi:10.1089/thy.2020.0322)
Supplement: Supplemental data [file Supp_TableS1.docx]

**Supplementary Table 1**

| Pt | BRAF | RAS | p53 | KM2D | TERT | PTEN | PBRM1 | NF1 | RB1 | MTOR | SMARC | other cancer associated mutations |
| --- | --- | --- | --- | --- | --- | --- | --- | --- | --- | --- | --- | --- |
| 1 | wt | wt | wt | wt | mut | mut | wt | wt | wt | wt | wt | SETD2  MEN1 |
| 2 | wt | NRAS Q61R | mut | mut | wt | wt | wt | wt | mut | wt | SMARCA4 | MEN1 |
| 3 | wt | wt | wt | wt | mut | mut | wt | wt | wt | wt | wt | RET |
| 4 | wt | NRAS  Q61R | wt | wt | wt | wt | wt | wt | wt | wt | wt | PIK3CA |
| 5 | wt | NRAS Q61R | mut | wt | wt | wt | wt | wt | wt | wt | wt | MSH6  PTCH1 |
| 6 | wt | NRAS G12V | mut | wt | wt | wt | wt | wt | wt | wt | wt |  |
| 7 | wt | wt | CN loss | wt | wt | wt | wt | wt | wt | wt | wt | EIF1AX  MAPK14 |
| 8 | wt | NRAS Q61R | mut. | wt | mut | wt | wt. | wt. | wt. | wt | wt |  |

Mutations identified by whole exome sequencing (7/8) and copy number (CN) changes identified by RNAseq in 7/8 ATC patients. Patient 8 received targeted sequencing for all 11 indicated genes.
