# Supplementary material for: Combination of Lenvatinib and Pembrolizumab Is an Effective Treatment Option for Anaplastic and Poorly Differentiated Thyroid Carcinoma
Source: Thyroid. 2021 Jul 8;31(7):1076–85. doi: 10.1089/thy.2020.0322 (PMC8290324; doi:10.1089/thy.2020.0322)
Supplement: Supplemental data [file Supp_TableS2.docx]

| **Pt Nr** | **Histological subtype** | **Surgical therapy** | **TNM at**  **diagnosis** | **Radiation**  **therapy/RAI** | **Chemotherapy regimen**  **before L/P** | **metastasis type /local relapse before L/P treatment start** |
| --- | --- | --- | --- | --- | --- | --- |
| 1 | PDTC (initially classified as ATC) | thyroidectomy (R2 resection) | pT4, N2, M1 (lung, bone), V2, L1, R2, G3 | - cervical hyperfractionated irradiation 39 Gy + doxorubicin  - radiation left femur and right ribs 6/7 (42 Gy)  - radiation me-diastinum 39 Gy | - 2 cycles cisplatin/doxorubicin  - 2 cycles carboplatin/paclitaxel | lung, bone, kidney, liver |
| 2 | ATC with spindle cell morphology | thyroidectomy (R2 resection), neck dissection stomach tube | pT4, N2, M1 (lung), V1, L1, R2 | cervical IMRT with 64,5 Gy + carboplatin/paclitaxel (3 cycles) | 5 additional cycles carboplatin/paclitaxel | lung, hilary/mediastinal lymph nodes, cervical relapse, |
| 3 | PDTC | thyroidectomy (R0) + neck dissection | pT3 pN1a (3/20) cM0 V1 L1 R0 G3 | ablative RAI 2 x  3,8 GBq (102 mCi)  7,5 GBq (203 mCi) |  | lung, bone, liver |
| 4 | ATC w/o differentiation | total thyroidectomy and central neck dissection | pT3a pN1a (2/21) L1 V1  M1 (lung) | brain metastasis IGRT 1 x 18 Gy with 70% isodose | 5 cycles paclitaxel weekly | lung, brain, cervical relapse |
| 5 | ATC with partial squamous cell differentiation | hemi-thyroidectomy left (R1), followed by total thyroidectomy and neck dissection | pT4a, pNx, L0, V1, Pn0  M0 R1 | cervical irradiation 70 Gy + carboplatin/paclitaxel | 5 cycles carboplatin/paclitaxel | lung, skin, bone, cervical relapse |
| 6 | ATC w/o differentiation | thyroidectomy, R2 resection | pT4b, pNx, pM1 (lung), V2 L1 R2 | cervical irradiation 60 Gy + doxorubicin |  | lung, cervical relapse |
| 7 | ATC + follicular thyroid carcinoma | thyroidectomy R2 resection | pT4, N1, M0, R0, V2, L0 | - RAI 3.8 GBq  - cervical IMRT 70 Gy | - CDPROO1X2101 trial Novartis (1 cycle PD-inhibitor)  - 5 cycles carboplatin/paclitaxel | lung, cervical relapse |
| 8 | ATC (spindle cell mor-phology) no differentiation | debulcing with R2 resection  stomach tube | pT4 pN1 L0 V1 M0 | IMRT 70 Gy + docetaxel /doxorubicin (3 months) | 3 cycles cisplatin/paclitaxel | lung, hilary lymph nodes, cervical relapse |

**Supplementary Table 2**

**Previous therapy and staging before L/P treatment**

RAI = radioactive iodine therapy, ATC = anaplastic thyroid carcinoma, PDTC = poorly differentiated thyroid carcinoma, IGRT = image guided radiotherapy, IMRT = intensity modified radiation therapy, RAI = radio iodine therapy
