# Supplementary material for: Combination of Lenvatinib and Pembrolizumab Is an Effective Treatment Option for Anaplastic and Poorly Differentiated Thyroid Carcinoma
Source: Thyroid. 2021 Jul 8;31(7):1076–85. doi: 10.1089/thy.2020.0322 (PMC8290324; doi:10.1089/thy.2020.0322)
Supplement: Supplemental data [file Supp_TableS3.docx]

**Supplementary Table 3**

**Criteria for response** **according to RECIST v1.1**

1. Evaluation of **Target Lesions**

**Complete  Response (CR):**

Disappearance of all target lesions. Any pathological lymph nodes   (whether target or non‐target) must have reduction in short axis to   < 10 mm.

**Partial  Response (PR):**

At least a 30% decrease in the sum of diameters of target lesions, taking as  reference the baseline sum of diameters.

**Stable Disease  (SD):**

Neither sufficient shrinkage to qualify for PR nor sufficient increase to qualify for PD,  taking as reference the smallest sum of diameters while on study.

**Progressive  Disease (PD):**

At least a 20% increase in the sum of diameters of target lesions, taking as reference  the smallest sum on study (this includes the baseline sum if that is the smallest on  study).  In addition to the relative increase of 20%, the sum must also demonstrate  an absolute increase of at least 5mm.

**Not Evaluable  (NE)**

Progression has not been documented and one or more target lesions have not  been assessed or have been assessed using a different method than baseline that  makes comparability impossible.

**2. Evaluation of Non‐target Disease:**

**Complete  Response (CR):**

Disappearance of all non‐target lesions.  All lymph nodes must be non‐pathological  in size (< 10 mm short axis).

**Non‐CR/Non‐PD**

Persistence of one or more non‐target lesion(s).

**Progressive  Disease (PD):**

Unequivocal progression of existing non‐target lesions.

**Not Evaluable  (NE)**

Progression has not been documented and one or more non‐target lesions have  not been assessed or have been assessed using a different method thanbaseline  that makes comparability impossible.

**3. Timepoint Response Evaluation:**

Timepoint Response: patients with target (+/‐ non‐target) disease.

Target Lesions   Non‐Target Lesions   New Lesions  **Timepoint Response**

CR  CR  No  **CRa**

CR  Non‐CR/Non‐PD  No  **PR**

CR  Not evaluated  No  **PR**

PR  Non‐PD or not all evaluated No  **PR**

SD  Non‐PD or not all evaluated No  **SD**

Not all evaluated   Non‐PD  No  **NE**

PD  Any  Yes or No   **PD**

Any  PD  Yes or No   **PD**

Any  Any  Yes  **PD**
