# Supplementary material for: Combination of Lenvatinib and Pembrolizumab Is an Effective Treatment Option for Anaplastic and Poorly Differentiated Thyroid Carcinoma
Source: Thyroid. 2021 Jul 8;31(7):1076–85. doi: 10.1089/thy.2020.0322 (PMC8290324; doi:10.1089/thy.2020.0322)
Supplement: Supplemental data [file Supp_TableS4.docx]

**Dierks Supplementary Table 4**

Pathologic response criteria after chemotherapy in solid tumors (Miller-Payne criteria (MPC))

Grade 1 NR = No response, No change or some alteration to individual malignant cells but no reduction in the overall cellularity

Grade 2 A minor loss of tumor cells but overall cellularity still high, up to 30% loss

Grade 3 between an estimated 30% to 90% reduction in tumor cells

Grade 4 more than 90% loss of tumor cells

Grade 5 pCR = pathologic complete response, No malignant cells identifiable in sections from the site of tumor
