# Supplementary material for: Combination of Lenvatinib and Pembrolizumab Is an Effective Treatment Option for Anaplastic and Poorly Differentiated Thyroid Carcinoma
Source: Thyroid. 2021 Jul 8;31(7):1076–85. doi: 10.1089/thy.2020.0322 (PMC8290324; doi:10.1089/thy.2020.0322)
Supplement: Supplemental data [file Supp_Data.docx]

**Individual patient histories**

Patient 1

Patient 1 was a 63 year old female initially diagnosed with ATC in 12/15. In 11/15 lung metastasis were observed via chest-CT performed due to a pneumonia. A diagnostic thoracoscopy showed an epitheloid tumor, with partial thyroglobulin expression. Staging revealed a thyroid tumor in the left thyroid lobe, and additional cervical lymph node metastasis, as well as bone metastasis (left femur, ribs 6/7 right). In 12/15 she underwent a thyroidectomy with R2 resection of the thyroid tumor. The tumor was initially classified as ATC pT4, N2, M1, V2, L1, R2, G3 (lung and bone metastasis). She received 2 cycles of doxorubicin (60 mg/m^2^) /cisplatin (120 mg/m^2^), which was not well tolerated by the patient. Then we performed cervical hyperfractionated radiation therapy 39 Gy (3 Gy per day), and additional irradiation of the left femur (42 Gy) and right ribs 6 and 7 (42 Gy) together with doxorubicin monotherapy as radiosensitizer. Due to increasing mediastinal lymph node metastasis she developed a SVC syndrome/obstruction and received then an additional irradiation of the mediastinum around the superior vena cava (36 Gy). From 05/16 to 06/16 she received 2 cycles of carboplatin/paclitaxel, but developed a pneumonia again and a severe neutropenia despite G-CSF support. A PET-CT showed tumor progression of lung- and bone metastasis, and she developed additional metastasis in the kidney. Histology was reassessed at the pathology department Freiburg, and the diagnosis was changed to **PDTC** instead of ATC. Despite partial thyroglobulin expressiony of the tumor tissue, the tumor showed no radioiodine uptake (RAI negative). After the disease progressed again (liver/lung/kidney/bone), and thyroglobulin levels raised to 2045 ng/ml, the lenvatinib (24 mg/day)/pembrolizumab regimen (L/P) was initiated in 06/16. Within four months, the patient achieved a radiologically assessed PR according to RECIST v1.1 criteria, and thyroglobulin dropped to 88 ng/ml. She was then clinically and radiologically stable for a total of two years, with thyroglobulin levels between 80 and 100 ng/ml. In total she received 34 cycles of pembrolizumab and received lenvatinib 24 mg for 24 months. Due to a knee surgery with infectious complications, she had to stop taking lenvatinib for one month (after taking it 24 months). During this time, the lung metastasis progressed and she developed new liver metastasis. Despite re-initiation of lenvatinib and addition of paclitaxel, she died due to disease progression 2 months later.

Patient 2

Patient 2 is a 76-year-old male, and was diagnosed with ATC with spindle cell morphology in 02/2016 (pT4, cN2, M1, V1, L1, R2). Thyroidectomy was performed achieving an R2 resection of the cervical tumor. Staging revealed cervical and mediastinal lymph node metastasis and pulmonary metastasis. The patient received irradiation therapy (IMRT 64.5 Gy) combined with 3 cycles of carboplatin/paclitaxel from 02/16 to 04/16. Due to dysphagia and extreme weight loss he received a stomach tube. Radiology assessment showed a stabilization of the disease, and he continued with 5 additional cycles of ´carboplatin AUC5/paclitaxel 200 mg/m^2^. In 10/16 the disease progressed with increasing lung metastasis, hilary and mediastinal lymph node metastasis and a cervical relapse, and the patient was switched to lenvatinib 24 mg/day and pembrolizumab 200 mg every 3 weeks. In 03/18, the CT showed a CR, which was confirmed by PET-CT 3 months later. Then lenvatinib was discontinued 21 months after L/P initiation, while pembrolizumab was continued until 01/20 (40 months total), and the patient is now in CR without any further therapy.

Patient 3

A 49-year-old female was diagnosed with PDTC in 05/16 including a large thyroid tumor and cervical lymph node metastasis (pT3 pN1a (3/20) cM0 V1 L1 R0 G3). She received a thyroidectomy and modified neck dissection, and the tumor could be completely removed (R0). In 06/16 and again in 10/16 she received an ablative radioiodine therapy, but was not responsive to the treatment and progressed 2 months later with cervical and mediastinal lymph node metastasis and new lung metastasis. Thyroglobulin increased to 586 ng/ml. In 12/16 the combination therapy of lenvatinib (24 mg) and pembrolizumab was started and the patient reached a radiologically assessed PR after 4 months of treatment, and thyroglobulin levels dropped to 6 ng/ml. She developed grade III adverse events with anorexia and abdominal pain, resulting in stepwise dose reduction of lenvatinib from 24 (1 month) to 20 (2 months), 14 (8 months) and 10 mg (3 months). Since even the lowest dose of 10 mg was not tolerated by the patient, we consequentially stopped the treatment after 14 months. As disease progression occured within a short time period again the patient was then treated with carboplatin/paclitaxel, followed by a paclitaxel/pembrolizumab regimen. Ten months after lenvatinib/pembrolizumab discontinuation, the regimen was reintroduced (lenvatinib 14 mg/pembrolizumab) and parenteral nutrition support as well as opioid treatment for abdominal pain was initiated. She nevertheless died due to disease progression 2 months later after re-initiation of the L/P treatment.

Patient 4

Patient 4 is a 68 year old female who presented with a large cervical tumor and lung metastasis at initial diagnosis in 05/17 (pT3a pN1a (2/21) L1 V1 M1 (lung) R0). She received a thyroidectomy (R0) and a central neck dissection showing an anplastic thyroid carcinoma without any differentiation. Then she received paclitaxel monotherapy from 05/17 to 10/17. Taxol monotherapy resulted in an initial stabilization of the disease (SD after 3 cycles), but after 5 months of therapy she progressed with new lung metastases and a solitary brain metastasis. After stereotactic irradiation of the brain metastasis (IGRT 1 x 18 Gy with 70% isodose), she was started on the lenvatinib (20 mg)/pembrolizumab regimen. A control PET/CT at 12 months after treatment initiation showed a PR according to RECIST v1.1 criteria, but all suspected pulmonal lesions were FDG-negative (CR according to EORTC PET response criteria). All pulmonary lesions were surgically removed and were devoid of viable tumor tissue. The brain metastasis was biopsied and was also devoid of viable tissue. Lenvatinib was stopped 14 months after treatment initiation. She is currently in CR 26 months after L/P initiation and receives pembrolizumab only.

Patient 5

Patient 5 was a 63 year old female with ATC diagnosed in 02/2018 with UICC stage pT4a, pNx, L0, V1, Pn0 . She was suspected to have a struma multinodosa with a fast growing node within the left thyroid. Therefore, she received a left-sided hemithyroidectomy, which showed an anaplastic thyroid carcinoma with partial squamous cell differentiation. The R1 resection was followed by a total thyroidectomy and neck dissection 3 weeks later. Then she received radiation therapy (70 Gy) combined with chemotherapy carboplatin/paclitaxel for 2 months. As swallowing was difficult she received a stomach tube. In 07/18 she developed a locoregional relapse of the cervical tumor, skin metastasis and bone metastases in several vertebral bodies (C6, T2 and T5), and consequently received 5 additional cycles of carboplatin/paclitaxel. Then she progressed with an increasing number of bone and additional lung metastases. Cervical disease progression and bilateral vocal cord paresis necessitated the insertion of a tracheostoma, and the therapeutic regimen was switched to lenvatinib (20 mg)/pembrolizumab in 09/18. She achieved a complete remission 10 months after initiation of the L/P treatment. One month later, while still on therapy, the tracheal tube was removed, but immediately following its removal the patient died from a massive cervical haemorrhage.

Patient 6

Patient 6 was an 88-year-old female diagnosed with ATC stage IVC with a huge cervical tumor in the left thyroid and lung metastasis diagnosed in 03/18 (pT4b, pNx, pM1, V2, L1, R2). She had been previously treated for autoimmune thyreoiditis. She received a hemithyroidectomy on the left side (R2), followed by a radiochemotherapy (60 Gy) with doxorubicin. As the cervical tumor relapsed within two months and lung metastasis progressed, she was started on the lenvatinib (14 mg)/pembrolizumab combination. After 3 months L/P, she had achieved a radiologically confirmed SD, but developed grade II/III AEs including anorexia, diarrhea, fatigue and oral mucositis resulting in treatment discontinuation 4 months after treatment start. She died due to disease progression 7 months after lenvatinib/pembrolizumab initiation.

Patient 7

A 63-year-old male was diagnosed with ATC and additional follicular thyroid carcinoma including cervical lymph node metastases in 08/17. After thyroidectomy/neck dissection and R0 resection of the cervical tumor (12 x 8 x 7 cm), he received one ablative radioiodine therapy (3.8 GBq), but developed a cervical relapse in 10/17. While receiving intensity modulated radiotherapy (IMRT) to his neck (60 Gy), he progressed with lung metastasis and new cervical lymph node metastasis. In 12/17 he was included in a clinical trial with an immune checkpoint inhibitor directed against PD1 (CPDROO1X2101 trial, 3 applications). He progressed within 6 weeks with new lung metastasis and a cervical relapse, was stopped in the trial and then received carboplatin/paclitaxel. After the first 3 cycles he had a PR, but then the dose of paclitaxel had to be reduced due to neurotoxic side effects, and the patient progressed again with lung metastasis and cervical progression. In 06/18 he was then switched to the lenvatinib (24 mg)/pembrolizumab regimen. After 3 months all metastasis and the cervical tumor regressed (PR according to RECIST v1.1). A control PET/CT in 06/19 showed a CR 12 months after L/P initiation. Lenvatinib was stopped in 09/19, and the patient continued with pembrolizumab monotherapy and is still in CR 19 months after L/P initiation.

Patient 8

A 59-year-old male was diagnosed with ATC with spindle cell morphology in 08/17 with a huge cervical tumor and lymph node metastasis (T4 N1 M0). R2 tumor debulcing was followed by radiochemotherapy with docetaxel and doxorubicin (3 months). New pulmonary metastasis was diagnosed 1 month later, and the patient then received three cycles of cisplatin 75 mg/m2 /paclitaxel 175 mg/m2. Despite chemotherapy, bilateral lung metastasis and hilary lymph node metastasis progressed and the cervical tumor relapsed. The patient stopped eating due to the huge cervical mass and received a stomach tube. Then the patient was switched to the lenvatinib (24 mg daily)/pembrolizumab treatment combination. Unfortunately, the patient died due to cervical tumor progression within 4 weeks after initiation of L/P therapy.
